# Reconstructing the Origin of Oxygenic Photosynthesis: Do Assembly and Photoactivation Recapitulate Evolution?

**DOI:** 10.3389/fpls.2016.00257

**Published:** 2016-03-02

**Authors:** Tanai Cardona

**Affiliations:** Department of Life Sciences, Imperial College LondonLondon, UK

**Keywords:** oxygenic photosynthesis, anoxygenic photosynthesis, photosystem, reaction center, water oxidation, photoactivation, photoassembly

## Abstract

Due to the great abundance of genomes and protein structures that today span a broad diversity of organisms, now more than ever before, it is possible to reconstruct the molecular evolution of protein complexes at an incredible level of detail. Here, I recount the story of oxygenic photosynthesis or how an ancestral reaction center was transformed into a sophisticated photochemical machine capable of water oxidation. First, I review the evolution of all reaction center proteins in order to highlight that Photosystem II and Photosystem I, today only found in the phylum Cyanobacteria, branched out very early in the history of photosynthesis. Therefore, it is very unlikely that they were acquired via horizontal gene transfer from any of the described phyla of anoxygenic phototrophic bacteria. Second, I present a new evolutionary scenario for the origin of the CP43 and CP47 antenna of Photosystem II. I suggest that the antenna proteins originated from the remodeling of an entire Type I reaction center protein and not from the partial gene duplication of a Type I reaction center gene. Third, I highlight how Photosystem II and Photosystem I reaction center proteins interact with small peripheral subunits in remarkably similar patterns and hypothesize that some of this complexity may be traced back to the most ancestral reaction center. Fourth, I outline the sequence of events that led to the origin of the Mn_4_CaO_5_ cluster and show that the most ancestral Type II reaction center had some of the basic structural components that would become essential in the coordination of the water-oxidizing complex. Finally, I collect all these ideas, starting at the origin of the first reaction center proteins and ending with the emergence of the water-oxidizing cluster, to hypothesize that the complex and well-organized process of assembly and photoactivation of Photosystem II recapitulate evolutionary transitions in the path to oxygenic photosynthesis.

## Evolution of reaction center proteins

Photochemical reaction centers are thought to have originated only once in the domain Bacteria. This is because currently there are no described strains in the domain Archaea with photosynthesis based on protein complexes containing chlorophyll or bacteriochlorophyll (Hohmann-Marriott and Blankenship, 2011). At the other end of the tree of life, eukaryotic algae and plants obtained photosynthesis via endosymbiosis of Cyanobacteria (Gould et al., 2008; Parfrey et al., 2010). Within Bacteria, there are currently seven phyla known to have strains with reaction centers, these are: Cyanobacteria, Chloroflexi, Firmicutes, Chlorobi, Proteobacteria, and those recently found in Acidobacteria (Bryant et al., 2007; Tsukatani et al., 2012) and Gemmatimonadetes (Zeng et al., 2014, 2015). Just a while ago, it was suggested that the phylum Actinobacteria might have been ancestrally capable of photosynthesis (Gupta and Khadka, 2016), as some strains in this phylum seem to have a vestigial chlorophyll synthesis pathway. Although a consensus on the type of bacteria in which photochemical reaction centers originated is lacking, it is understood that both Type I and Type II reaction centers have a common origin. This conclusion is supported by the structural similarities of the core proteins, the relative positions of the pigments in the reaction center, and commonalities in the first photochemical steps during charge separation (Olson and Pierson, 1987; Nitschke and Rutherford, 1991; Cardona et al., 2012; Cardona, 2015). Furthermore, it is also clear that oxygenic photosynthesis originated in a lineage of bacteria that was ancestral to the phylum Cyanobacteria (Cardona et al., 2015), as Photosystem II is exclusively found within members of this group and was only transferred to photosynthetic eukaryotes at a later stage.

The story of photosynthesis began early in the history of life. When exactly? It is not yet known with certainty. Organic carbon found in rocks 3.8 billion years old has some signatures of possible photoautotrophy (Rosing, 1999; Nisbet and Fowler, 2014). It is generally accepted that photosynthesis was well established from at least 3.5 billion years ago (Butterfield, 2015; Knoll, 2015). Evidence is growing for a redox-stratified ocean 3.2–3.0 billion years ago, suggestive of some oxygenic photosynthesis (Crowe et al., 2013; Planavsky et al., 2014; Satkoski et al., 2015) and it is likely that Cyanobacteria were already flourishing around the Great Oxygenation Event 2.4 billion years ago (Lyons et al., 2014). But, is the molecular evolution of the photochemical machinery consistent with the geochemical record? Could photochemical reaction centers and chlorophyll synthesis have emerged as early as 3.8 billion years ago? And if they did, what does it imply for the earliest forms of life? Could the water-oxidizing complex of Photosystem II have really appeared already 3.2 billion years ago?

**Figure 1:**
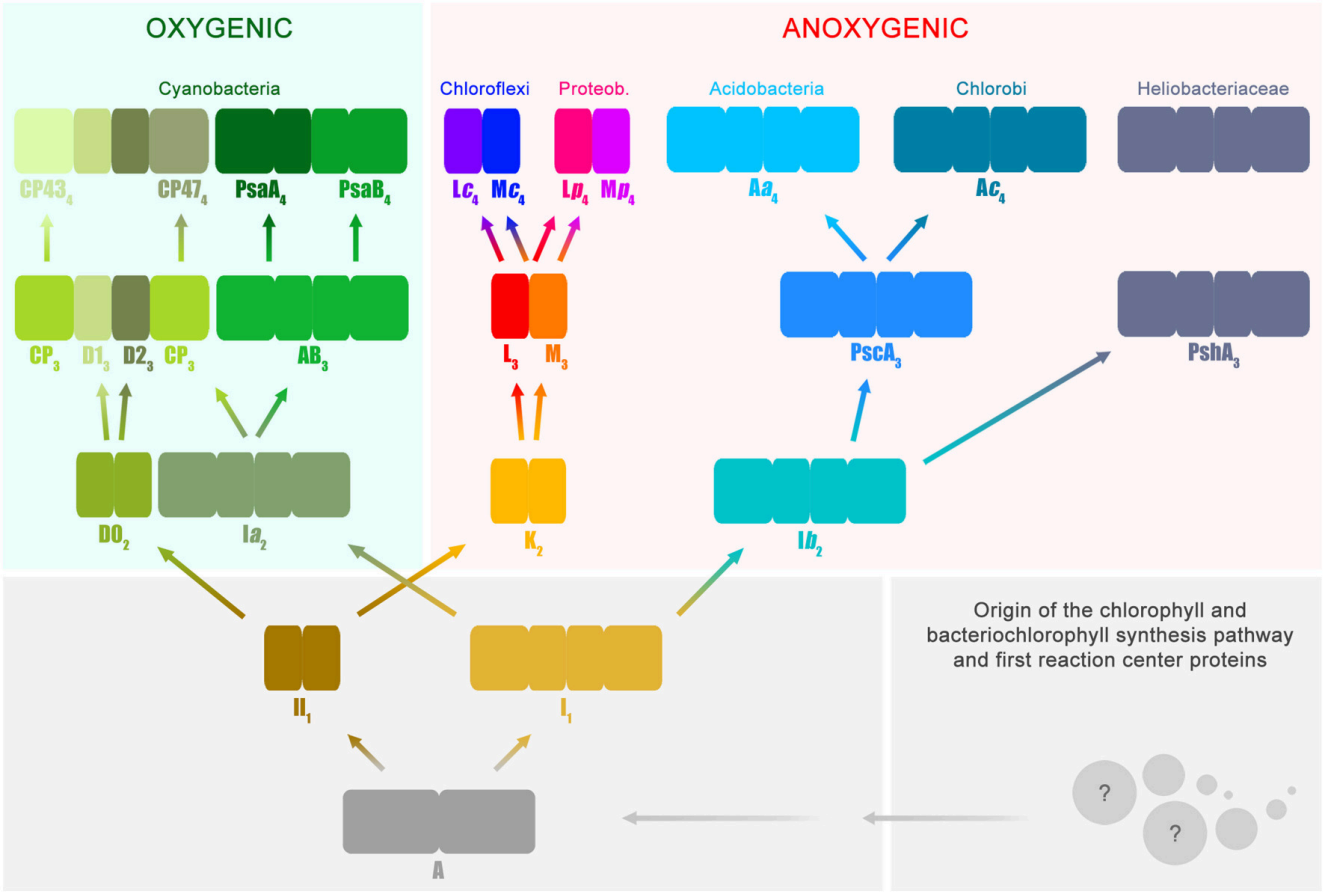
**Evolutionary relations of reaction center proteins**. This scheme is based on the well-known phylogenetic relationships of reaction center proteins, which I have reviewed in detail before (Cardona, 2015). At the bottom right, the spheres with question marks represent the earliest evolutionary events that led to the evolution of the chlorophyll and bacteriochlorophyll synthesis pathway and the first reaction center proteins. It has been suggested that reaction center proteins might have originated from single-helix pigment-binding proteins (Allen and Vermaas, 2010) or from proteins related to the respiratory Cytochrome *b* proteins (Xiong and Bauer, 2002). Both hypotheses merit further consideration. The ancestral reaction center protein **A** gave rise to two new classes of proteins ancestral to Type I (**I**_**1**_) and II (**II**_**1**_), respectively. It is highly likely that these early stages of reaction center evolution occurred at a time well before the radiation of modern bacterial forms. Therefore, it is difficult to envision the mechanisms by which the ancestral reaction center evolved into two new forms and the evolutionary forces that aided such divergence. The subscript indicates transitional stages away from ancestral protein **A**. Both **I**_**1**_ and **II**_**1**_ separated into two new classes of proteins, one lineage led to the evolution of Photosystem I and II, employed in oxygenic photosynthesis and today only found in the phylum Cyanobacteria and photosynthetic eukaryotes. The other lineage led to the type of reaction center proteins employed by anoxygenic phototrophic bacteria. Type I reaction centers (longer rectangles) are characterized by having 11 transmembrane helices: the first 6 helices are the antenna domain in charge of light harvesting and the last 5 helices are the reaction center domain in charge of photochemistry. In Type II reaction centers (smaller rectangles) the first 6 helices are missing, and only the reaction center domain is found, the last 5 helices. Photosystem II is unique because it is associated with antenna proteins, the CP43 and CP47 subunits, which originated from a Type I reaction center. The nature of the ancestral reaction center protein **A** is uncertain, was it more like a Type I or Type II reaction center? Did it have an iron-sulfur cluster or a non-heme iron? Did it have an antenna domain or was this fused later with a reaction center protein to make the first Type I reaction centers? At the moment, from the existing structural and sequence data, it is not possible to answer these questions with certainty. Reaction center **A** probably had some traits from each reaction center type and also some unique traits no longer present in reaction center proteins today.

Based on functional and structural homology, and in combination with phylogenetic analysis, it is possible to deduce several stages in the history of photosynthesis with reasonable confidence. I will introduce now a slightly unconventional notation that will facilitate the accurate description of these evolutionary stages (Figure 1); or simply put, I will give names to key ancestral reaction center proteins. This will help to highlight the correct position of Photosystem II within the entire diversity of reaction center proteins. Nevertheless, I will only and exclusively use this notation to refer to evolutionary transitions.

All reaction center proteins have a common origin (Olson, 1981; Nitschke and Rutherford, 1991; Sadekar et al., 2006; Cardona, 2015). This means that the evolutionary history of all reaction center proteins can be traced back to a single ancestral protein at the dawn of photosynthesis. I will refer to this primordial reaction center protein as **A** in Figure 1. It is worth noting here that the appearance of the first reaction center capable of photochemistry and made of protein **A** already implies a long and complex evolutionary process. This is because the earliest evolutionary stages required the origin of a complete pathway for pigment synthesis coupled to the emergence of the first reaction center protein that could be assembled into a functional photochemical machine. At a later stage and driven by as yet unknown evolutionary pressures, the ancestral reaction center diverged into two new forms. One form was ancestral to all Type I reaction centers and a second form was ancestral to all Type II reaction centers.

All Type I reaction center proteins share among each other significantly more sequence and structural homology than with any Type II protein. In other words, they share a more recent common ancestor with each other than with any Type II. The same is true the other way around, all Type II reaction center proteins share among each other significantly more sequence and structural homology than with any Type I protein, so they descended from a common Type II ancestral protein. I will refer to the ancestral Type I reaction center as **I**_**1**_ and the ancestral Type II reaction center as **II**_**1**_(Figure 1). The subscript indicates that they are one transitional step away from **A**. This “transitional step” may represent a gene duplication event, horizontal gene transfer within ancestral populations of bacteria, or speciation. The evolutionary path from the primordial reaction center to the ancestral forms of Type I and Type II reaction centers was also very complex; very drastic genetic and structural rearrangements are required to explain their current differences. However, a detailed description of these changes is outside the scope of the present paper, but I have discussed some of these before (Cardona, 2015 and reference therein). In here, it should suffice to say that an ancestral reaction center, by some mechanism, gave rise to two new types. At this point it is tempting to ask: was the primordial protein **A**, Type I or Type II? This question is perhaps unanswerable. But a better question to ask is, what traits present in Type I and Type II reaction centers today were also present in the most ancestral reaction center? I will highlight a few of these conserved traits between the two types throughout the text below.

The early divergence of Type I and Type II reaction center is clear from sequence and structural comparisons; see Sadekar et al. (2006) for example. This straight-forward, often overlooked, early divergence of reaction center types helps solve one of the longest-standing questions on the origin of photosynthesis. In which of the groups of phototrophic bacteria did photosynthesis first evolve? Did it evolve in the phylum Chlorobi or Chloroflexi, or within the Heliobacteriaceae family of Firmicutes, or perhaps in Cyanobacteria? The answer is: none of them. This is because the known groups of phototrophs are crown groups and none of them carry the ancestral protein **A**, nor **I**_**1**_, nor **II**_**1**_. The groups of bacteria that carried these ancestral proteins belong to stem groups, very likely no longer part of the current biodiversity and extinct for several billion years.

At a later stage, protein **II**_**1**_ diverged into two new distinct reaction center proteins: I will refer to these as **D0**_**2**_ and **K**_**2**_. **D0**_**2**_ is ancestral to both D1 and D2, the core subunits of Photosystem II found only in the phylum Cyanobacteria. **K**_**2**_ is ancestral to both L and M, the core subunits of anoxygenic Type II reaction centers. Using the subscript to denote the transitional stages away from the primordial protein **A**, then D1 and D2 are equivalent to **D1**_**3**_ and **D2**_**3**_, because they are predated consecutively by **D0**_**2**_, **II**_**1**_, and **A**. Similarly, the ancestral L and M are equivalent to **L**_**3**_ and **M**_**3**_. However, unlike D1 and D2, L and M undergo another evolutionary transition. As two new phyla of bacteria containing an anoxygenic Type II reaction center appeared, the phylum Chloroflexi and the phylum Proteobacteria, L and M diverged. Therefore, the L and M found in phototrophic members of the phylum Chloroflexi form a well-defined subgroup and the L and M found in phototrophic members of the phylum Proteobacteria form a different subgroup (Deisenhofer et al., 1984; Ovchinnikov et al., 1988a,b; Beanland, 1990; Cardona, 2015). I will thus refer to the set found in Chloroflexi as **L***c*_**4**_ and **M***c*_**4**_ and to the set found in Proteobacteria as **L***p*_**4**_ and **M***p*_**4**_. In relatively recent evolutionary time, some bacteria belonging to the phylum Gemmatimonadetes obtained a Type II reaction center via horizontal gene transfer from a gammaproteobacterium (Zeng et al., 2014, 2015). It is also possible that some bacteria from the phylum Firmicutes, of the genus *Alkalibacterium*, obtained Type II reaction centers from a gammaproteobacterium (Perreault et al., 2008), but this needs experimental verification.

Now, the last common ancestor of the phylum Cyanobacteria had already **D1**_**3**_ and **D2**_**3**_ because the earliest diverging strains of the genus *Gloeobacter* have a standard Photosystem II. It follows then that the phototrophic organism that carried the ancestral **D0**_**2**_ protein is not part of the current diversity and predated the last common ancestor of the phylum Cyanobacteria. In a similar way, all phototrophic members of the phylum Proteobacteria carry well-defined **L***p*_**4**_ and **M***p*_**4**_ suggesting that all L and M subunits found in photorophic Alpha-, Beta-, and Gammaproteobacteria originated from those in an ancestral proteobacterium carrying **L***p*_**4**_ and **M***p*_**4**_, already distinct from **L***c*_**4**_ and **M***c*_**4**_. The same reasoning applies to Chloroflexi. Following along the same lines, the organism that contained the anoxygenic Type II reaction center composed of the ancestral proteins **L**_**3**_ and **M**_**3**_ is not part of the current described biodiversity. The existence of this ancestral bacterium must predate the arrival of phototrophy to the phylum Chloroflexi and the phylum Proteobacteria. In consequence, it is impossible that Cyanobacteria obtained Type II reaction center proteins via horizontal gene transfer from a phototroph of the phylum Chloroflexi or Proteobacteria. The evolutionary relationship among Type II reaction center proteins excludes any scenario where D1 and D2 originated from **L***p*_**4**_, **M***p*_**4**_, **L***c*_**4**_, **M***c*_**4**_, or their ancestral forms **L**_**3**_ and **M**_**3**_. This clearly shows that Photosystem II and anoxygenic Type II reaction centers have followed independent evolutionary pathways since the earliest stages of photosynthesis. So, even though Photosystem II looks, at first glance, a lot more sophisticated than anoxygenic Type II reaction centers; the complex itself retains uncanny ancestral characteristics. Some of these will be described further below. The relationship between Photosystem II and anoxygenic Type II reaction centers is fundamental to understand the origin of oxygenic photosynthesis, because some of the protein motifs required to coordinate the Mn_4_CaO_5_ cluster might have been inherited from the ancestral Type II reaction center protein, **II**_**1**_.

In parallel to the evolution of Type II reaction centers, the ancestral protein to all Type I reaction center proteins, **I**_**1**_, also diversified into two forms: **I***a*_**2**_ and **I***b*_**2**_. On the one hand, **I***a*_**2**_ gave rise to two new reaction center proteins, named in Figure 1 as **AB**_**3**_ and **CP**_**3**_. The former is ancestral to both Photosystem I reaction center proteins, PsaA and PsaB; and the latter is ancestral to the antenna proteins of Photosystem II, CP47 and CP43. In this case, this set of proteins is four transitional stages away from the primordial reaction center protein **A**, and therefore they can be written as **PsaA**_**4**_, **PsaB**_**4**_, **CP47**_**4**_, and **CP43**_**4**_. Like D1 and D2, these set of proteins are found exclusively within today's cyanobacterial diversity. The detailed evolution of the CP47 and CP43 antenna proteins will be discussed in the next section.

On the other hand, **I***b*_**2**_ is ancestral to all Type I reaction center proteins present only in anoxygenic phototrophic bacteria. **I***b*_**2**_ gave rise to two new proteins: **PshA**_**3**_ that today is only found in the family Heliobacteriaceae of the phylum Firmicutes and **PscA**_**3**_. In turn, **PscA**_**3**_ undergoes another evolutionary transition as the phylum Chlorobi and Acidobacteria diversified. Thus, I shall call them **A***c*_**4**_ for Chlorobi and **A***a*_**4**_ for Acidobacteria. The **A***c*_**4**_ and **A***a*_**4**_ reaction center proteins share a common ancestor, **PscA**_**3**_, but they are phylogenetically distinct from each other displaying unique sequence variations and structural characteristics (Bryant et al., 2007; Tsukatani et al., 2012). Thus, it cannot be said that the reaction center from Acidobacteria was obtained via horizontal gene transfer from a phototrophic bacterium belonging to the phylum Chlorobi, or vice versa.

Comparable to Type II, the evolutionary relations of Type I reaction center proteins exclude the possibility that Cyanobacteria obtained Photosystem I from a bacterium of the phylum Chlorobi, Acidobacteria, or from a heliobacterium. This is because **PsaA**_**4**_ and **PsaB**_**4**_ are predated by **AB**_**3**_, and not **A***a*_**4**_, **A***c*_**4**_, or **PshA**_**3**_. In other words, **A***a*_**4**_, **A***c*_**4**_, and **PshA**_**3**_ share among each other significantly more sequence and structural homology than with **PsaA**_**4**_, **PsaB**_**4**_, or with **CP43**_**4**_ and **CP47**_**4**_. The last common ancestor of **A***a*_**4**_, **A***c*_**4**_, and **PshA**_**3**_ is **I***b*_**2**_ (Figure 1), and accordingly it can be concluded that the organism that had **I***b*_**2**_ must have existed before the evolutionary radiation that gave origin to the bacteria containing **PscA**_**3**_ and **PshA**_**3**_ at a later evolutionary stage. This implies that the reaction center proteins that gave rise to Photosystem I and the antenna proteins of Photosystem II also started to diverge very early during the evolution of photosynthesis, in a similar fashion to Type II reaction center proteins.

## Evolution of the CP43 and CP47 subunits and their interaction with D1 and D2

The evolution of the antenna proteins of Photosystem II has not been dealt with in as much detail as the core reaction center proteins. Mostly because it is assumed that the CP43 and CP47 subunits of Photosystem II originated from the division or truncation of a gene encoding a Type I reaction center protein, thus leaving only the first six transmembrane helices of the antenna domain (Mix et al., 2005). Nonetheless, a thorough examination of the antenna proteins and the way they are connected with the reaction center core opens a new window to the fascinating evolutionary events that were at play during the early stages of photosynthesis.

The CP43 and CP47 proteins are connected with D1 and D2 via two peripheral chlorophyll *a* molecules known as Chl_Z_ and Chl_D_, also referred to as ChlZ_D1_ and ChlZ_D2_. The chlorophylls are coordinated by D1-H118 and D2-H117 (**Figures 3A,B**) and mediate excitation energy transfer from the CP43 and CP47 antenna to the reaction center core (Lince and Vermaas, 1998; Vasil'ev and Bruce, 2000). From an evolutionary standpoint, the connection of the antenna with the core proteins is of particular importance because homologous peripheral chlorophylls are also present in Photosystem I coordinated by homologous histidine ligands. Sequence comparisons show that this histidine is also present in PshA and the PscA of the Chlorobi and are likely to coordinate a bacteriochlorophyll molecule in order to mediate excitation transfer from the antenna domain to the core domain (Baymann et al., 2001; Cardona, 2015). The presence of these peripheral pigments in Type I reaction centers proteins and in Photosystem II, as well as the remaining sequence identity in this region of the core proteins, strongly suggest that they were present in the primordial reaction center protein, **A** (Figure 1). In other words, the peripheral chlorophylls of Photosystem II, Chl_Z_, and Chl_D_, are ancestral traits retained since the origin of the first reaction centers. It suggests that the primordial Type II reaction center made of protein **II**_**1**_—at the dawn of photosynthesis—was interacting closely with the antenna domain of a Type I reaction center protein. This is consistent with two distinct reaction centers side-by-side, in the same membrane, early during the evolution of photosynthesis in at least some ancestral phototrophic bacteria predating the origin of water oxidation, and likely as well, the diversification of most phyla containing phototrophic bacteria today. The idea of two reaction centers evolving within the same organisms has been hypothesized before; see for example Olson (1981) or Allen (2005). In addition, recent genomic and phylogenetic analyses have provided some support to this as well (Sousa et al., 2013; Cardona, 2015; Harel et al., 2015).

**Figure 2:**
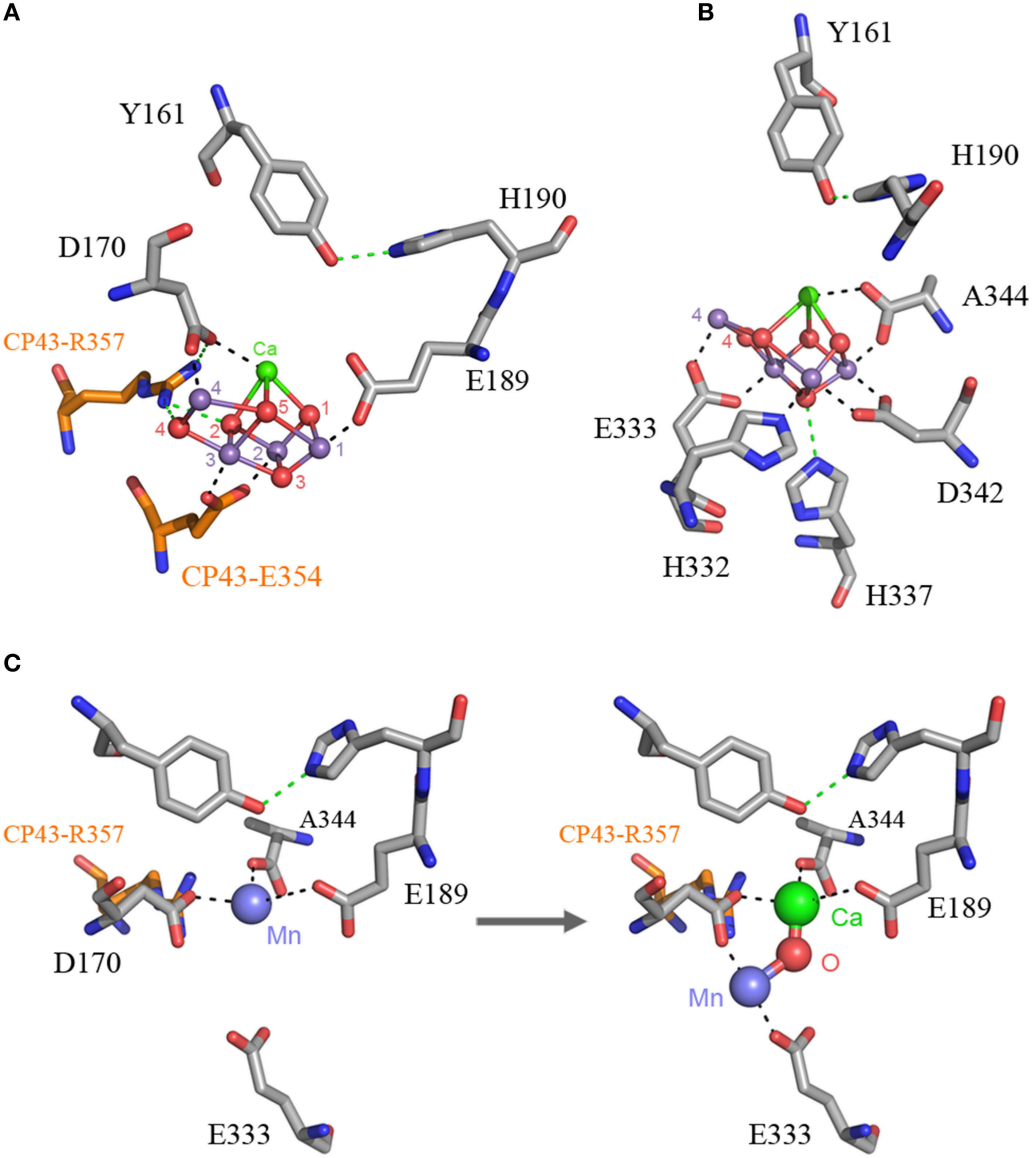
**The Mn_4_CaO_5_ cluster of Photosystem II as resolved in the crystal structure by Umena et al. (2011), PDB ID: 3WU2**. Panel **(A)** shows the cluster coordinated by the inner ligands D170 and E189 and the ligands provided by the CP43 subunit, E354, and R357. Panel **(B)** shows the ligands provided from the C-terminus of the D1 protein. Panel **(C)** shows a proposal for the high-affinity Mn binding site based on evolutionary grounds and supported by mutagenesis and spectroscopy (see text). After oxidation of the first Mn(II) to Mn(III), which might occur concomitantly with the deprotonation of a ligating water molecule, Ca^2+^ binds. The binding of Ca^2+^ shifts the initially bound Mn(III) to a position similar to that of Mn4 in the intact cluster.

**Figure 3:**
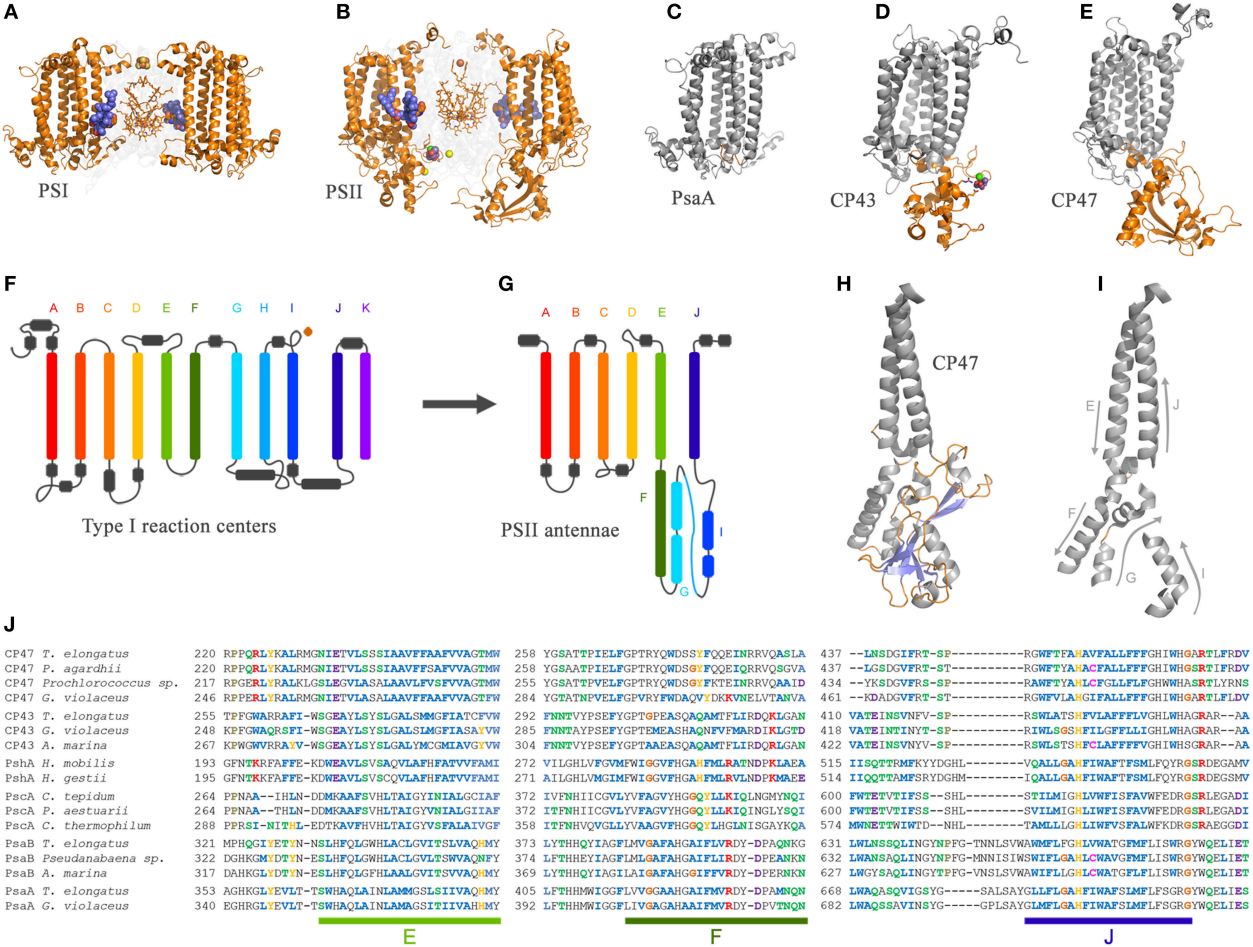
**Comparison of the antenna domain of Photosystem I reaction center proteins and the antenna proteins of Photosystem II from the crystal structures PDB ID: 1JB0 and 3WU2 from *Thermosynechococcus* spp., respectively**. **(A,B)** highlight the core antenna of Photosystem I and II respectively (orange). Blue spheres highlight the conserved peripheral chlorophylls that allow excitation energy transfer from the core antenna to the reaction center, named Chl_Z_ and Chl_D_ in Photosystem II. **(C–E)** highlight the differences between the antenna domain of the PsaA subunit of Photosystem I with that of the CP43 and CP47, respectively. Note that the extrinsic domain is absent in PsaA. Panels **(F,G)** show and schematic representation of a Type I reaction center and a Photosystem II antenna protein. The alpha-helices have been color coded to show the homologous positions. Panels **(H,I)** show detail of the folding pattern of the extrinsic domain from the CP47 subunit. Panel **(J)** shows a sequence alignment of all Type I reaction center proteins including the CP47 and CP43 subunits. Only three fragments are shown for clarity, those corresponding to transmembrane helices E, F, and J in Type I reaction centers and their respective matching sequences in the antenna proteins of Photosystem II. The sequence alignment was performed using Clustal Omega that applies a Hidden Markov Model-based algorithm (Sievers et al., 2011). The full alignment is available on request.

The origin of water oxidation mandates the presence of both types of reaction centers within the same bacterium. Not just because of the energetic requirements of shuttling electrons from water to NADP^+^, but also because the CP43 antenna protein of Photosystem II, which originated from a Type I reaction center protein, is involved in the coordination of the water-oxidizing complex. Residue E354 of the CP43 coordinates Mn3 and Mn2 of the Mn_4_CaO_5_ cluster and R357 offers a hydrogen bond to O2 and O4 (Ferreira et al., 2004; Umena et al., 2011), see Figure 2. Site-directed mutants of these two residues show a severe impairment of the water oxidation cycle and fail to grow photoautotrophically (Ananyev et al., 2005; Hwang et al., 2007; Strickler et al., 2008; Shimada et al., 2009; Service et al., 2011). In addition to this, both CP43 and CP47 play important roles in water and proton access into or out of the cluster (Umena et al., 2011; Linke and Ho, 2013). This close interaction between the antenna proteins and the water-oxidizing complex is possible thanks to a large extrinsic lumenal domain between the 5th and 6th transmembrane helices of both the CP47 and CP43 proteins (Figure 3). The extrinsic domain has about 130 amino acids in CP43 and about 190 in CP47, in both cases showing remarkable structural similarities (Kamiya and Shen, 2003). The assumption that the CP43 and CP47 proteins originated from a fission or truncation of a Type I reaction center gene presupposes that the extrinsic domain originated as a sequence insertion on the ancestral antenna protein, denoted **CP**_**3**_ in Figure 1. This is because no such extrinsic domain is present in the antenna part of any of the Type I reaction center proteins described to date. However, in-depth sequence and structural comparisons reveal that the antenna proteins of Photosystem II most likely emerged from the remodeling of an entire Type I reaction center protein and not from a partial gene duplication (Figures 3F–J). While the first five transmembrane helices are indeed homologous between the antenna and the reaction center proteins; the 6th helix in Type I reaction centers (helix F) is homologous to an alpha-helix in CP43 and CP47 located outside the membrane, in the extrinsic domain, and follows immediately after the 5th helix (Figures 3F–J). In this way, what was thought to be the 6th transmembrane helix of CP43 and CP47, actually corresponds to the 10th helix (J) in Type I reaction centers. Consequently, the CP43 and CP47 proteins originated from an entire Type I reaction center protein that, over time, underwent a transformation, where the entire region spanning the 6th to the 9th transmembrane helices (F to I) protruded outside the membrane. This change could have been triggered by relatively few mutations such that the 6th helix became unstable within the membrane. Hypothetically, the changes could have enhanced energy transfer to the ancestral Type II reaction center by allowing a better docking of the antenna domain with the Type II core and thus conferred a selective advantage. At the same time, the extrinsic domain was now free to interact with the donor side of the early D1 and D2 proteins fostering perhaps the oxidation of aqueous Mn(II) and the origin of the Mn_4_CaO_5_ cluster.

Similar structural changes have been accomplished in site-directed mutants, where the insertion of a transmembrane helix or the topology of a membrane protein is radically altered by a single or few amino acid substitutions (Hessa et al., 2007; Seppälä et al., 2010). Some membrane proteins that undergo similar dramatic topological changes under physiological conditions have been described before (Von Heijne, 2006). What is more, these kind of evolutionary transitions are not completely foreign to photosynthesis. For example, the PsbO subunit of Photosystem II, which is located outside the membrane toward the lumen, might have originated from an outer-membrane protein related to porins (De Las Rivas and Barber, 2004; Iverson, 2006). Also, the FMO light-harvesting complex found in Chlorobi and Acidobacteria is proposed to have originated from the refolding of PscA (Olson and Raymond, 2003).

## The ancient recruitment of additional protein subunits

**Figure 4:**
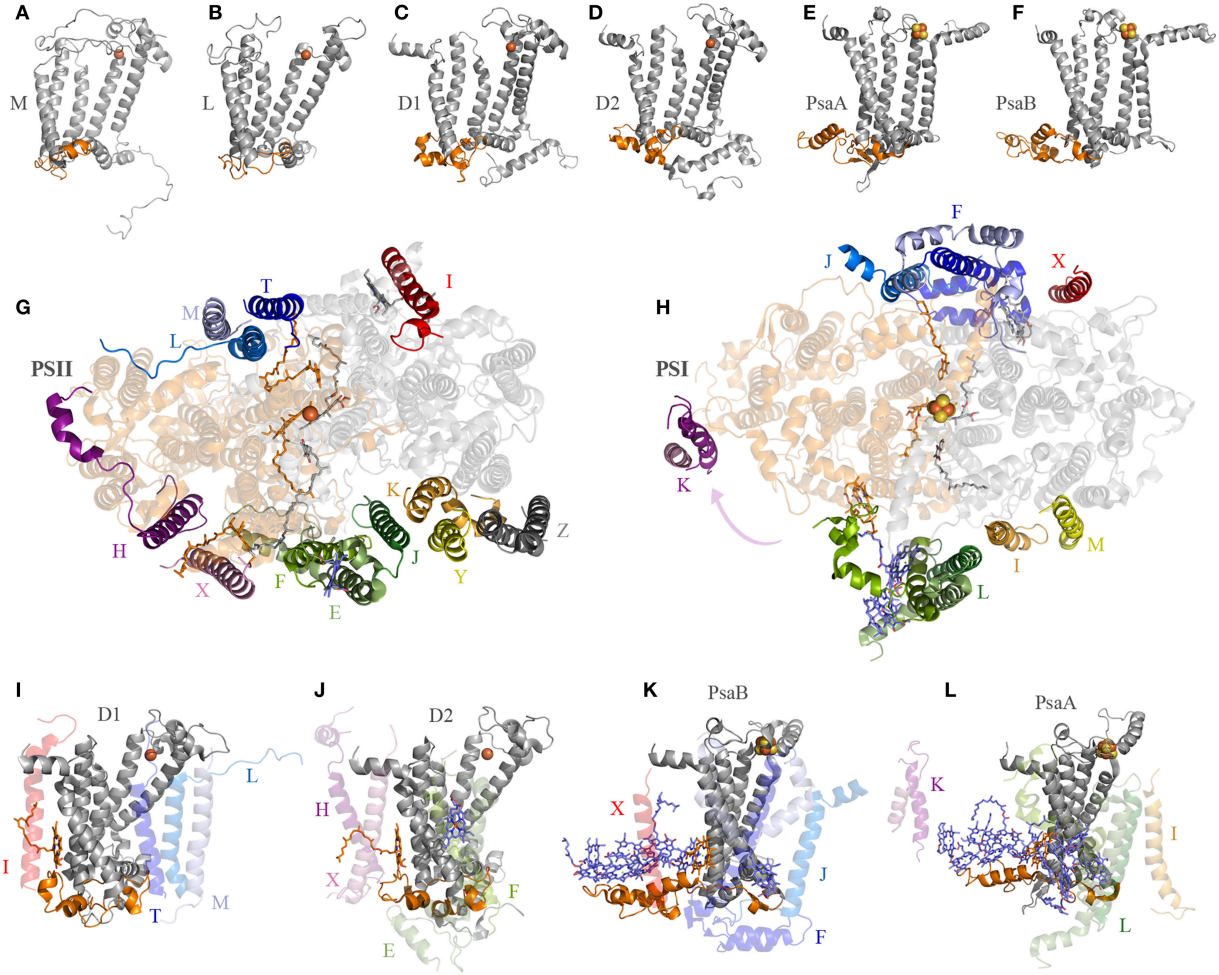
**Interaction of reaction center proteins and the small peripheral subunits**. Panels **(A–F)** show the reaction center proteins of the anoxygenic Type II reaction center of *Blastochloris viridis* (M and L, PDB ID: 2PRC), *Thermosynechococcus vulcanus* (D1 and D2, PDB ID: 3WU2), and *Thermosynechococcus elongatus* (PsaA and PsaB, core domain only, PDB ID: 1JB0). The orange colored cartoon highlights the protein segment found between the 1st and 2nd transmembrane helices of the core proteins (7th and 8th in Photosystem I). Panels **(G,H)** show the position of the peripheral subunits of Photosystem II and Photosystem I. Panels **(I–L)** show the interaction of the core proteins with some of the small subunits and nearby pigments.

One big difference between Photosystem II and anoxygenic Type II reaction centers is that Photosystem II is made of many protein subunits. In addition to D1, D2, and the antenna proteins, it requires at least 13 extra proteins as seen in the crystal structures (Zouni et al., 2001; Kamiya and Shen, 2003). The entire collection of subunits can vary from species to species and from Cyanobacteria to photosynthetic eukaryotes (Pagliano et al., 2013): it includes the Cytochrome *b*_559_, a range of small membrane proteins located at the periphery of the complex (Figure 4), added to the extrinsic polypeptides that shield and stabilize the Mn_4_CaO_5_ cluster. Like Photosystem II, Photosystem I is also adorned with a range of peripheral small subunits (Jordan et al., 2001; Mazor et al., 2014, 2015). A structural comparison of Photosystem II and Photosystem I shows that the peripheral small subunits are arranged around the core proteins in a strikingly similar pattern (Figures 4G,H). In Photosystem II, the Cytochrome *b*_559_ (PsbE and PsbF) together with PsbJ, which in total make three transmembrane helices, are located perpendicular to the Q_A_-Fe^2+^-Q_B_ axis. At an equivalent position in Photosystem I the PsaL subunit binds the core proteins perpendicular to A_1A_-F_X_-A_1B_: PsaL has three transmembrane helices. At the opposite side of the Q_A_-Fe^2+^-Q_B_ axis in Photosystem II, there are three single-helix subunits PsbL, PsbM, and PsbT making a three-helix bundle. In Photosystem I, PsaJ, and PsaF are found at an equivalent position. While PsaJ has a single transmembrane helix, PsaF has two helices. Although, the second helix of PsaF does not span the entirety of the membrane, because it is bent in the middle (Jordan et al., 2001; Mazor et al., 2014, 2015). Interestingly, the homodimeric reaction center found in phototrophic Chlorobi is known to bind two Cytochrome *c*_551_ proteins. Cytochrome *c*_551_ has three transmembrane helices and it is the direct electron donor to P840 (Oh-oka et al., 1995). It can be predicted that each Cytochrome *c*_551_ binds symmetrically to the reaction center in a position equivalent to that of Cytochrome *b*_559_-PsbJ and PsbLMT in Photosystem II, or PsaL and PsaFJ in Photosystem I. In fact, this is the only position where Cytochrome *c*_551_ could bind so that the cytoplasmic domain, which contains the heme, can be positioned near P840 on the periplasmic side of the membrane. This location is also consistent with recent cross-linking experiments done with isolated reaction centers (He et al., 2014). Are these incredible cases of molecular convergent evolution or was the ancestral reaction center to Photosystem I and Photosystem II more complex than previously anticipated? I should reiterate here that the ancestral reaction center to Photosystem I and Photosystem II is the primordial reaction center **A**, ancestral to all reaction centers (Figure 1).

D1 and D2 are distinguished from the L and M subunits by the presence of three additional protein segments (Cardona, 2015), these are: (1) an extension of the amino acid sequence in between the 1st and 2nd transmembrane helices (Figures 4A–D); (2) an extension between the 4th and 5th transmembrane helices; and (3) an extension beyond the 5th transmembrane helix at the C-terminus. These three differences are tied to the functional and structural differences between the anoxygenic Type II reaction center and Photosystem II (Cardona, 2015). These three protein segments are present in both D1 and D2, suggesting that they must have been in the ancestral protein to both, identified as **D0**_2_ in Figure 1, before the evolution of the Mn_4_CaO_5_ cluster. From these three segments, the extension between the 1st and 2nd transmembrane helices is essential for the binding of some of the small subunits of Photosystem II (Figure 4). In D1, this loop provides a binding site to PsbI and PsbL. In D2 the loop provides a binding site to the Cytochrome *b*_559_ and PsbX. It appears that this segment's main function is to provide a site for protein-protein interactions with additional subunits. It follows then that before the origin of water oxidation and before the divergence of D1 and D2, the nascent Photosystem II was already interacting with additional protein subunits of some sort, which are not found in anoxygenic phototrophs containing Type II reaction centers. In fact, like D1 and D2, PsaA and PsaB also have an extension of the protein sequence in between the equivalent transmembrane helices 7th and 8th (homologous to 1st and 2nd in Type II) that interacts with PsaL and PsaFJ, see Figure 4. Additionally, in the cross-linking experiment mentioned above, the PscA subunit from the reaction center of *Chlorobaculum tepidum* cross-linked with the Cytochrome *c*_551_ via a lysine also found in between the 7th and 8th helices (He et al., 2014). All in all, it is a strong indication that the primordial reaction center **A** was interacting with membrane bound proteins, regardless of their original function.

## Origin of the Mn_4_CaO_5_ cluster

Several hypotheses regarding the origin of the Mn_4_CaO_5_ cluster have been proposed before. One of them suggested that the tetramanganese cluster evolved from the interaction of an anoxygenic Photosystem II with a manganese catalase (Raymond and Blankenship, 2008). In this case, the dinuclear Mn cluster of catalase was somehow transferred to Photosystem II. An interaction with a second catalase, should have donated the second pair of Mn ions. A second hypothesis proposed that the cluster originated from natural Mn oxide precipitates present in the ocean (Sauer and Yachandra, 2002). A third hypothesis proposed that the ancestral Photosystem II used bicarbonate as the direct electron donor before the use of water, and this was complexed with Mn (Dismukes et al., 2001). Fortunately, with the great surge in genomic and structural data from Cyanobacteria, it is now possible to reconstruct the origin of the catalytic cluster at a level of detail uncommon for other metalloenzymes. Phylogenetic and structural analysis of the D1 protein of Photosystem II showed that some of them appeared to have diverged at different stages during the evolution of the Mn_4_CaO_5_ cluster (Cardona et al., 2015). The different types of D1, listed from the earliest to the latest diverging groups, are:

- An atypical D1 sequence found in the genome of *Gloeobacter kilaueensis* JS-1 (Saw et al., 2013; Cardona et al., 2015)
- Group 1: a type of D1 associated with chlorophyll *f* -producing cyanobacteria, also known as super-rogue D1 (Murray, 2012; Gan et al., 2014)
- Group 2: a type of D1 expressed in the night or in darkness, also known as rogue D1 or sentinel D1 (Murray, 2012; Wegener et al., 2015)
- Group 3: a type of D1 expressed under low-oxygen conditions, also known as D1' (Summerfield et al., 2008; Sicora et al., 2009)
- Group 4: the dominant form of D1 expressed under normal conditions and found in all Cyanobacteria and photosynthetic eukaryotes. This group also includes the so-called “high-light” forms of D1.

The common trait of the earliest evolving forms of D1, including the unusual sequence from *Gloeobacter kilaueensis*, Group 1, and Group 2, is that all of them are missing ligands to the Mn_4_CaO_5_ cluster (Murray, 2012; Cardona et al., 2015). I will call these forms of early evolving D1, “atypical sequences” or “atypical D1 forms.” On the other hand, the latest evolving D1 forms, those of Group 3 and Group 4, have a complete set of ligands to the cluster. I will refer to these two groups as “standard sequences” or “standard D1 forms.” It is therefore tempting to suggest that when the atypical sequences appeared for the first time, the Mn_4_CaO_5_ cluster had not evolved yet to its standard form. Only the standard form of D1, those of Group 4, has been characterized in detail. Unfortunately, the function of all other forms of D1 remains quite poorly understood and somewhat mysterious, but they might confer advantages under particular environmental circumstances, such as under anaerobic conditions (Wegener et al., 2015) or challenging light conditions (Gan et al., 2014). It is important to note that the function of these early evolving D1 forms now might not be the same as when they first evolved.

**Figure 5:**
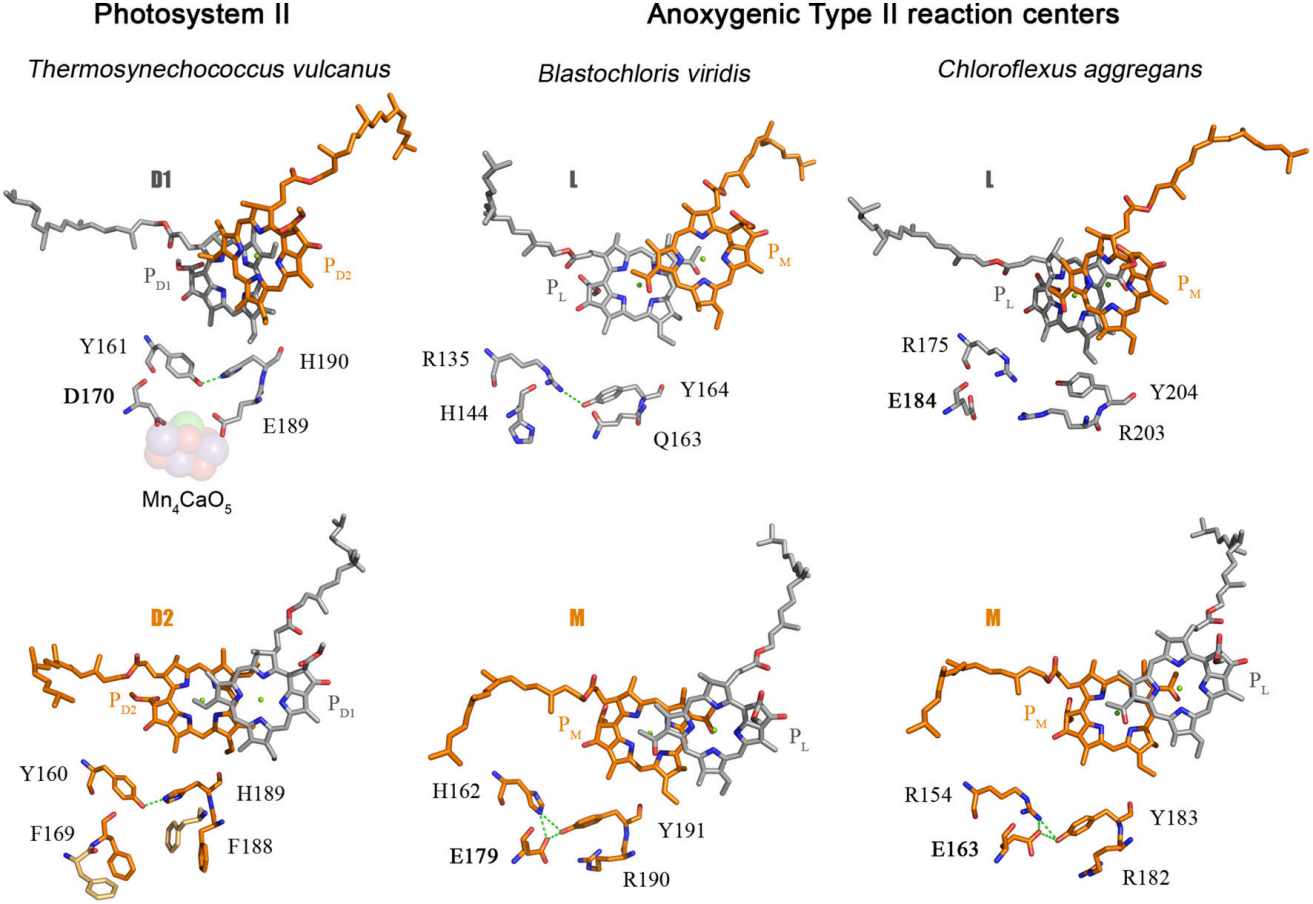
**Comparison of the electron donor sides of Type II reaction centers from *Thermosynechococcus vulcanus* (PDB ID: 3WU2), with those from *Blastochloris viridis* (PDB ID: 1JB0), and *Chloroflexus aggregans***. The highlighted residues are at strictly homologous positions as determined by sequence and structural comparisons; the variation in number is due to the fact that different reaction center proteins are of different sizes in different strains of bacteria. The L and M from *Chloroflexus aggregans* are homology models made with the SWISS-MODEL service, automated mode, using the structure of the *Blastochloris viridis* reaction center as a template.

The D1 protein of Photosystem II provides seven ligands to the Mn_4_CaO_5_ cluster. These can be divided in two groups: (1) D170 and E189, which are located in the CD loop between the 3rd and 4th helix; and (2) the ligands located in the C-terminal lumenal extension beyond the 5th transmembrane helix, H332, E333, H337, D342, and A344. Then, how did the ligand sphere around the Mn_4_CaO_5_ cluster evolve? The first ligand to have appeared was a glutamate at position equivalent to aspartate 170 (D170) of the crystal structures from *Thermosynechococcus vulcanus* (Figure 2). This is because there is a glutamate at this position in both the L and M subunits of the Chloroflexi and in the M of the Proteobacteria. There is also a glutamate at this position in some of the early branching forms of the D1 protein, see Figure 5 and Cardona et al. (2015). This suggests that the ancestral Type II reaction center protein, **II**_**1**_, probably had a glutamate at this position.

The ancestral Type II reaction center protein is also likely to have had a C-terminal extension after the 5th transmembrane helix that folded into an alpha-helix located near the donor side (Cardona, 2015). This is because the L subunit found in strains of the genus *Roseiflexus* and in some Proteobacteria also have a C-terminal extension that share sequence homology with the terminal alpha-helix present in D1 and D2. Surprisingly, all the Type I reaction center sequences found in phototrophic Chlorobi also have a C-terminal extension that is predicted to fold into an alpha-helix. It raises the intriguing possibility that a C-terminal alpha-helical extension is a trait original from the primordial reaction center **A**.

Based on these comparisons, it can be deduced that the ancestral Type II reaction center protein, **II**_**1**_, already had some of the basic components that at a later stage would become essential in the coordination of the Mn_4_CaO_5_ cluster: a glutamtate at position 170 and an alpha-helical domain extension at the C-terminus. I should reiterate here that **II**_**1**_ is ancestral not only to D1 and D2, but also to L and M, and therefore the ancestral bacterium that carried this protein almost certainly existed before the phyla Cyanobacteria, Chloroflexi, or Proteobacteria came into existence.

As I discussed before, the ancestral Type II reaction center protein, **II**_**1**_, diverged into two new forms: **D0**_**2**_ and **K**_**2**_. In the evolutionary transition from **II**_**1**_ to **D0**_**2**_, in a lineage that would later give rise to the phylum Cyanobacteria, a new feature was gained. This is the tyrosine-histidine pair located at positions homologous to 161 and 190 of the D1 in the crystal structures (Umena et al., 2011). These have been retained in all D1 sequences as the Y_Z_-H190 pair and in D2 as Y_D_-H189. Thus, a triad made of Tyr-Glu-His was formed in **D0**_**2**_ and it probably helped maintain the correct folding of the protein via a network of hydrogen bonds (Figure 5). A similar triad has evolved independently in the M subunit of bacteria of the genus *Blastochloris*, but rather than having a tyrosine at a position equivalent to 161, it has a histidine, making a pair with a tyrosine nearby. That triad in the M subunit of *Blastochloris* has no redox role and it seems to be only of structural importance. At this stage the Type II reaction center made of **D0**_**2**_ was still anoxygenic, but the triad was already present in both monomers of the reaction center. Then a major evolutionary transition occurred to the organism containing **D0**_**2**_ that changed the chemistry of the reaction center. This evolutionary innovation allowed the reaction center to oxidize the tyrosine in the triad upon charge separation, resulting in the formation of the tyrosyl radical. In other words, the oxidizing power of the “special pair” cation, P^•+^, increased.

The redox potential of P${}_{\text{D}1}^{•+}$/P_D1_ in standard Photosystem II is around 1200 mV (Rappaport et al., 2002; Ishikita et al., 2006), the redox potential of Y${}_{\text{Z}}^{•}$/Y_Z_ is calculated to be near 970 mV and that of Y${}_{\text{D}}^{•}$/Y_D_ near 760 mV (Vass and Styring, 1991). If we assume that the potential for P^•+^/P in the ancestral Photosystem II before water oxidation was similar to that in Chloroflexi or Proteobacteria, which is measured to be between 360 (Bruce et al., 1982; Collins et al., 2009) and 500 mV (Moss et al., 1991; Williams et al., 1992), respectively. Then, the potential of P^•+^/P in the ancestral Photosystem II had to increase by about 300–400 mV to at least be able to oxidize a tyrosine with the properties of Y_D_. In fact, a mutant reaction center from *Rhodobacter sphaeroides* with the potential of P870^•+^/P870 up-shifted to at least 800 mV can oxidize a tyrosine (Kalman et al., 1999) and exogenous Mn(II) (Thielges et al., 2005). Then how did P^•+^ become so oxidizing in the ancestral Photosystem II before water oxidation was possible?

Ishikita et al. (2006) calculated the influence of cofactors and protein charges on the redox potential of P_D1_. In comparison with the anoxygenic Type II reaction center from *Rhodobacter sphaeroides*, the use of chlorophyll *a* instead of bacteriochlorophyll *a* contributes 160 mV. Up to 200 mV are attributed to the effect from atomic charges and the protein dielectric volume of the antenna proteins and the small subunits. The presence of the Mn_4_CaO_5_ cluster up-shifts the potential another 200 mV. Finally, the side chains around P_D1_ tune down the potential by about −135 mV; see Cardona et al. (2012) for a full discussion. If we consider that the ancestral Photosystem II made of **D0**_**2**_ started with a low potential special pair, then just the acquisition of the antenna proteins and some additional peripheral subunits, combined with the use of chlorophyll instead of bacteriochlorophyll, could have up-shifted the potential by at least 360 mV, possibly more. This could have been enough to trigger the oxidation of the tyrosine located on both sides of the reaction center coupled to Mn(II) oxidation. The presence of glutamate near the tyrosine implies that upon oxidation of Mn(II) to Mn(III), the carboxyl side-chain of E170 could have coordinated and partially stabilized the metal. In this scenario, aqueous Mn(II) seems like a plausible electron donor to photosynthesis in an ancestral bacterium prior to the evolution of proper water oxidation. What is more, the initial oxidation and binding of Mn could have pushed the potential of P^•+^/P even further up, allowing additional oxidation steps or the binding of extra Mn ions. From this evolutionary perspective, it is therefore unnecessary to invoke a catalase or naturally occurring inorganic Mn oxide precipitates as precursors to the Mn_4_CaO_5_ cluster. It is also unnecessary to suggest that bicarbonate was a transitional direct electron donor before water. Aqueous Mn(II) might have been the direct electron donor once tyrosine oxidation was possible, followed in time, by water.

The second ligand to evolve was glutamate at position 189. The earliest evolving form of D1, the atypical sequence of *Gloeobacter kilaueensis*, only has a glutamate at position 189 and lacks all other ligands. This sequence is unique among D1 proteins because it has retained numerous ancestral traits (Cardona et al., 2015). In particular, the C-terminal alpha-helix looks like D2 and has retained some residues conserved in D2, but no longer in any other D1. In addition, none of the ligands at the C-terminus are present. Judging by the predicted structural similarities with the C-terminus of D2 and its phylogenetic position, it is highly likely that at the evolutionary stage when this sequence branched out, these ligands had not appeared yet. This implies that glutamate 170 and 189 predated the appearance of the ligands at the C-terminus. Another clue about the origin of the first ligands is found within D2: unlike D1, both glutamate ligands changed to phenylalanines (F169 and F188) and their presence impedes the binding of metals near Y_D_-H189. No such phenylalanines are present in any other Type II reaction center protein and therefore it seems they evolved to minimize the possibility of metal oxidation on the D2 side. This suggests that a glutamate at position 189 was also likely in **D0**_**2**_ and was lost in D2 on the path to heterodimerization of the reaction center.

The existence of an ancestral homodimeric Photosystem II capable of oxidizing manganese in each monomer has been hypothesized before (Rutherford and Nitschke, 1996; Rutherford and Faller, 2003; Williamson et al., 2011; Fischer et al., 2015). While the molecular evidence is somewhat compelling; it is not possible to tell with certainty whether such a reaction center could assemble a metal center of some sort or not, such as a mononuclear or dinuclear Mn cluster. If that early catalytic cluster did exist, it is also very difficult to deduce what kind of chemistry it carried out and whether it could have performed some partial water oxidation, even if inefficiently. Hypothetically, this early photosystem could have trapped at least two Mn atoms, which could have been oxidized sequentially from Mn(II) to Mn(IV) and thereby accumulating four positive charges. During photoactivation experiments, in the absence of Ca^2+^, oxidation of Mn(II) to Mn(III) occurs for several turnovers without the trapping of any Mn(IV); however, in mutants where D170 was changed to E170, as in the ancestral photosystem, Mn(II) was trapped and oxidized to Mn(IV) and possibly even as a dinuclear Mn_**2**_(IV,IV) complex (Campbell et al., 2000). It was suggested that E170 could provide a Mn binding site with a less positive reduction potential for the Mn(III)/Mn(IV) couple (Campbell et al., 2000). In addition, if Mn is complexed with bicarbonate this could lower its redox potential below 700 mV making it accessible to the early reaction center (Kozlov et al., 1997; Dismukes et al., 2001). It is not inconceivable that the early Photosystem II, before the divergence of D1 and D2, may have been able to assemble a simpler catalytic cluster. This early cluster could plausibly catalyze the partial oxidation of water to peroxide or, alternatively, the inefficient oxidation of water to oxygen.

At this stage the ligands at the C-terminus started to evolve. In the diversity of D1 proteins some of these ligands start to appear for the first time in Group 1 and Group 2 forms, suggesting that such ligands were never present in the equivalent D2 side. The appearance of the ligands at the C-terminus may have been selected in order to retain the metals near Y_Z_-H190: at first so that the cluster could be reassembled more quickly, and later to retain the cluster for several turnovers before falling apart. At this stage, oxygen concentrations in the environment or within the cell were likely extremely low. Thus, the formation of reactive oxygen species would have been also very low and so the high turnover rates of D1, as seen in standard Photosystem II today, were probably not needed. However, as water oxidation became more efficient and optimized, so photoprotective mechanisms should have become more sophisticated and specialized.

It is likely that any transitional Mn cluster and its early chemistry could have benefited from controlled deprotonation reactions, as the formation of di-μ-hydroxo or di-μ-oxo bridges might have resulted in the release of protons (Dau and Haumann, 2008). In fact, the atypical sequence from *Gloeobacter kilaueensis* and Group 1 D1 sequences have what appear to be proton exit pathways via the Y_Z_-H190 and toward the lumen. The proton pathways, both at the donor and acceptor side, start to resemble those in standard forms of D1 only in Group 2, even though these sequences lack ligands at the C-terminus. At the evolutionary stage that led to Group 1 and Group 2 sequences branching out, it is possible that a more sophisticated cluster already existed. Group 2 sequences can be found having either E170 or D170, so at some point in the evolutionary transition from Group 1 to Group 3, D170 is preferred over E170, possibly to optimize the shape of the cluster (Cardona et al., 2015).

Group 3 D1 sequences, or the low-oxygen form of D1, have all of the features required for water oxidation and a Photosystem II carrying this type of D1 can assemble a cluster and oxidize water (Sugiura et al., 2012). An experimental characterization of Photosystem II carrying one of these forms of D1 showed that the complex seemed to be slightly impaired at certain stages during the catalytic cycle, at least when tested under ambient oxygenic concentrations (Sugiura et al., 2012). It is possible that this type of D1 was predominantly in use when the oxygen concentrations in the environment and within the cell were still very low. The transition from Group 3 to Group 4 might have consisted only of fine-tuning the core of Photosystem II to run in a more oxidizing environment.

## Assembly intermediates of photosystem II may represent evolutionary transitions

Levy et al. (2008) suggested that the evolution of multiprotein complexes can be viewed as the sequential assembly of these complexes over a long period of time. From this perspective the starting point in the evolution of oxygenic photosynthesis is a simple anoxygenic Type II reaction center and culminates with the complex water-oxidizing enzyme we know today, with each new layer of complexity built upon the other. The implication of this is that the key evolutionary transitions that led to the appearance of water oxidation may be preserved in the assembly of the protein complex and in the process of photoactivation of the Mn_4_CaO_5_ cluster.

The assembly of Photosystem II is modular and a highly organized process (Komenda et al., 2012; Nickelsen and Rengstl, 2013). At the earliest stage of assembly, the D1 protein binds PsbI and separately D2 binds the Cytochrome *b*_559_. Then these two modules come together to make what looks like a primitive reaction center, composed of the two core subunits, a cytochrome, a small subunit, and devoid of antenna proteins, the Mn_4_CaO_5_ cluster, and the extrinsic polypeptides (Komenda et al., 2004; Dobáková et al., 2007). I have shown now how the earliest Type II reaction center made of **II**_**1**_ was probably interacting with additional subunits of some sort via a protein extension located between the 1st and 2nd transmembrane helix, which in Photosystem II serves as the place for protein-protein interactions with—specifically—PsbI and the Cytochrome *b*_559_. Once the early reaction center made of **D0**_**2**_ developed a special pair capable of oxidizing tyrosine, it is expected that the oxidation of Mn becomes possible. Somewhat intriguingly, it has been suggested that at this early stage of assembly, Mn is preloaded into the system via an assembly factor termed PratA in Cyanobacteria (Stengel et al., 2012). This occurs before the C-terminus of D1 is completely processed, suggesting that at this stage complete photoactiavtion is not possible. This early stage in biogenesis could mimic an ancestral metal-binding photosystem before the origin of the tetramanganese cluster.

Separately, CP43 forms a subcomplex with at least PsbK and PsbZ; and CP47 makes a subcomplex with PsbH, PsbL, and PsbT. The antenna subcomplexes then bind to the D1-D2-Cytochrome *b*_559_-PsbI reaction center to form a complete Photosystem II monomer (Sugimoto and Takahashi, 2003; Boehm et al., 2011), but still lacking the completely assembled cluster. Only after this stage can photoactivation of the Mn_4_CaO_5_ cluster occur. Plausibly mirroring evolution, the Mn_4_CaO_5_ cluster could have only evolved after the antenna proteins were associated with the reaction center, as the CP43 protein provides ligands to the cluster. I have also mentioned how Photosystem I binds numerous additional subunits that interact with the reaction center in a way very similar to Photosystem II. It is possible then, that many of these subunits were recruited quite early during the origin of the first reaction centers. If the antenna proteins of Photosystem II evolved from a Type I reaction center protein that was interacting with additional subunits, then it is not surprising that CP43 and CP47 bind a series of small polypeptides even before associating with D1 and D2. Upon photoactivation and relatively late in biogenesis, the extrinsic polypeptides bind the lumenal side of Photosystem II to isolate and stabilize the Mn_4_CaO_5_ cluster.

The first ligand to the Mn_4_CaO_5_ cluster to have evolved was likely a glutamate at position 170, followed by glutamate 189. This is paralleled during the process of photoactivation, as the first Mn(II) is oxidized to Mn(III) and bound to the high-affinity Mn binding site, known to be in part provided by D170 (Nixon and Diner, 1992; Campbell et al., 2000; Asada and Mino, 2015). The oxidation of Mn(II) to Mn(III) is accompanied by a deprotonating event of one of the ligating water molecules (Dasgupta et al., 2008). This is considered to be the first intermediate, which is unstable until the binding of Ca^2+^ followed by an uncharacterized conformational change (Tamura and Cheniae, 1987; Tamura et al., 1989; Chen et al., 1995; Tyryshkin et al., 2006). Beside D170 being part of the high affinity binding site, Dasgupta et al. (2007) suggested that the first bound Mn may also be coordinated by a N-donor ligand and speculated to be H332 or H337. Because these two histidines are too far from D170 to bind the same Mn, doubt was cast on the role of D170 as part of the high-affinity binding site (Becker et al., 2011). However, from an evolutionary perspective one would expect the first Mn to be bound by D170 and E189. If this is true, it can be predicted that the high-affinity binding site is located in a position near to the Ca^2+^ in the fully assembled cluster. In this position the first bound Mn is coordinated by D170, E189, and A344; the N-donor ligand detected by electron spin echo envelope modulation spectroscopy may be due to the presence of R357 from the CP43 subunit (Figure 2C). Although, R357 might appear counterintuitive as a N-donor ligand, arginine-metal interactions are not uncommon in metalloproteins; and arginine is known to ligate Mn in arginase (Di Costanzo et al., 2006). This position is not completely inconsistent with the distance measured by Asada and Mino (2015) using pulsed electron-electron double resonance spectroscopy considering that in the absence of the cluster the ligand sphere should be somewhat shifted. It is also consistent with a six-coordinate tetragonally-elongated or a five-coordinate square-pyramidal geometry as measured using EPR (Campbell et al., 2000). Upon Ca^2+^ binding, the uncharacterized conformational change is due to Ca^2+^ shifting the position of Mn and taking its correct position. Mn(III) then moves to a position similar to that of Mn4 in the crystal structure, where it is coordinated by D170 and E333. This is also consistent with the work by Cohen et al. (2007) that showed E333 mutants were impaired in the binding of the first Mn. After the binding and oxidation of Mn(II) to Mn(III), and the subsequent binding of Ca^2+^, a second Mn(II) is oxidized to Mn(III), which quickly leads to a fully assembled Mn_4_CaO_5_ cluster. Unfortunately, the fast events after the binding of the second Mn remain to be characterized; however, the evidence for an intermediary photoactivation step made of two Mn and involving at least D170 and E333 seems to be strong (Dasgupta et al., 2008; Becker et al., 2011).

## Concluding remarks

How long did it take for the ancestral Photosystem II to evolve the Mn_4_CaO_5_ cluster? Let us remember that the ancestral reaction center made of a double **D0**_**2**_ could have performed some kind of metal-centered catalysis and even perhaps some inefficient water oxidation. An answer to this question can be calculated by modeling the rates of evolution of D1 and D2 from the known phylogenetic relationships of Cyanobacteria and photosynthetic eukaryotes. Assuming that the last common ancestor of Cyanobacteria lived before the Great Oxygenation Event (2.4 billion years ago); then, the D1 and D2 divergence may have started more than a billion years before it. Even such early dates require rates of D1 and D2 evolution almost two orders of magnitude higher than those observed since the last common ancestor to the phylum Cyanobacteria.

**Figure 6:**
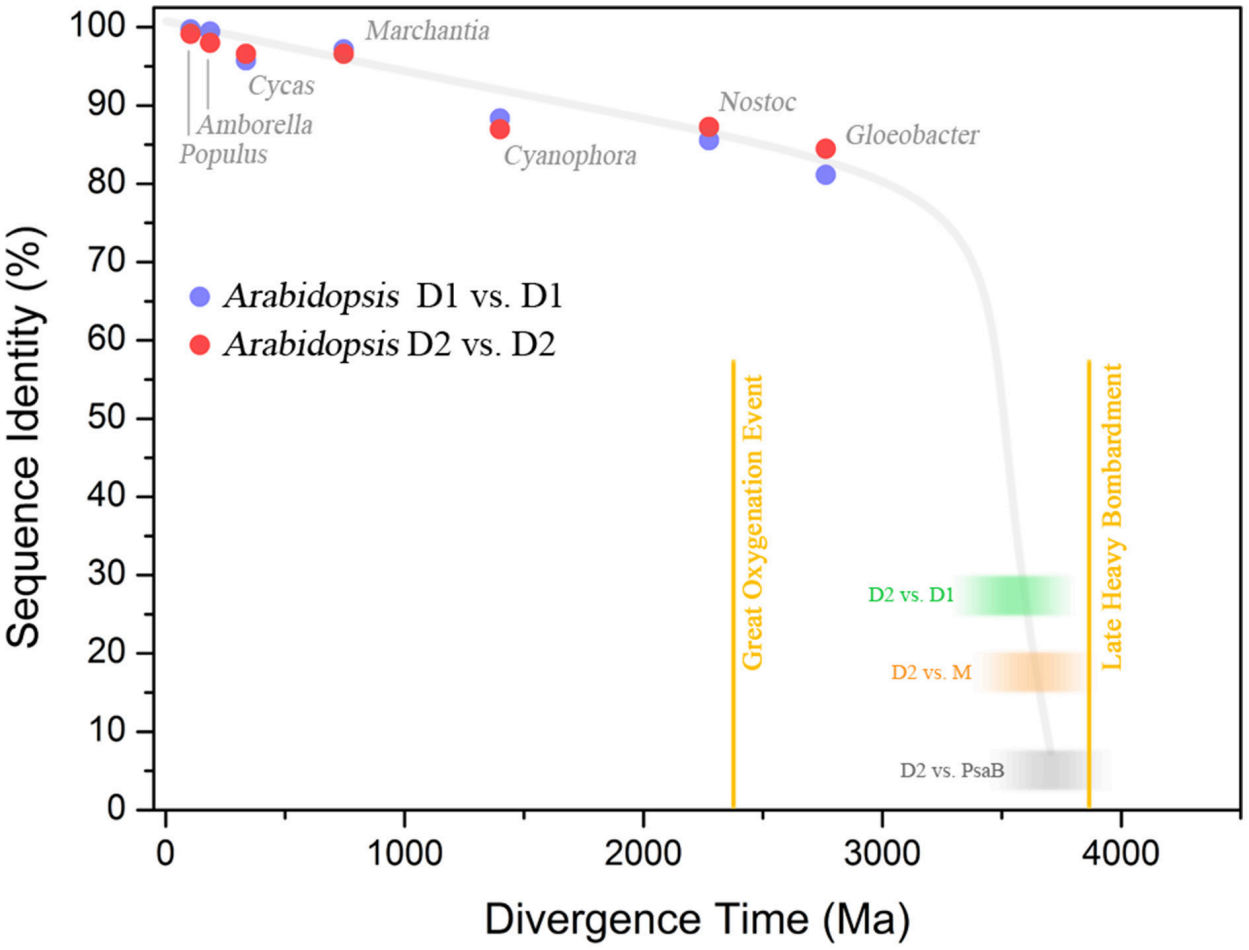
**Sequence identity of Photosystem II reaction center proteins as a function of time**. The blue dots show the percentage of sequence identity between the D1 protein sequence of *Arabidopsis thaliana* compared to that in other organisms. The red dots show the percentage of sequence identity of D2. The green blurred box shows the level of sequence identity between D1 and D2 of *Arabidopsis* and the blur represents uncertainty. The orange box shows the sequence identity of D2 compared to the M subunit of *Chloroflexus aurantiacus.* The gray box shows the sequence identity of D2 compared to the PsaB subunit of Photosystem I from *Thermosynechococcus elongatus*. This was calculated by overlaying the 3D structures of D2 and PsaB proteins and counting the conserved residues (the identity is below 5%), see Cardona (2015). The plot shows that D1 and D2 have been changing at an almost constant rate since the Great Oxygenation Event (GOE) around 2.4 billion years ago. It also highlights that the events that led to the divergence of Type I from Type II reaction centers (gray box), the anoxygenic from oxygenic Type II reaction centers (orange box), and D1 from D2 (green box), must have occurred very early in the history of life. It is also likely that these early events in the evolution of photosynthesis probably occurred relatively fast after the origin of the first reaction center protein. The divergence times in plant evolution were taken from Clarke et al. (2011). The time for *Cyanophora paradoxa*, representing the origin of plastids, was assumed to be in between 1.1 and 1.5 Ga (Butterfield, 2000; Yoon et al., 2004). The time for *Nostoc* sp. PCC 7120, representing the origin of heterocystous Cyanobacteria, was assumed to have occurred after the GOE (Golubic et al., 1995; Tomitani et al., 2006). The divergence of the genus *Gloeobacter violaceus* sp. PCC 7421 was assumed to have occurred before the GOE. The divergence time for D1/D2, D2/M, and D2/PsaB are hypothetical, but in order to explain the origin and diversification of photosynthesis within the age constraints of planet Earth, very fast rates of evolution are needed at the earliest stages. It suggests that the earliest stages of Photosystem II evolution, such as the divergence of D1 and D2, might have occurred soon after the origin of photochemical reaction centers.

Consider the following example. The standard D1 protein of *Gloeobacter violaceus* and *Arabidopsis thaliana* share 81.6% sequence identity. *Gloeobacter* and *Arabidopsis* are separated by more than two billion years of evolution. In contrast, the D1 and D2 proteins of *Gloeobacter* share between each other only 33.3% identity over the entire length of the sequences, while the D1 and D2 of *Arabidopsis* share 25.7% identity. *Gloeobacter* is the earliest branching genus of Cyanobacteria and it is reasonable to think that it branched out before the Great Oxygenation Event (Criscuolo and Gribaldo, 2011; Schirrmeister et al., 2013; Shih et al., 2013). This implies that when *Gloeobacter* branched out D1 and D2 were already highly divergent. If we assume a constant rate of evolution, the divergence time for D1 and D2 would be placed well before the formation of the planet, which makes no sense (Figure 6). Now, when modeling the evolution of reaction center proteins, not only do we need to account for the D1 and D2 divergence, but also the divergence of the LM-branch of Type II reaction center proteins, and the divergence of Type I reaction centers; all of which predated the split of D1 and D2 (Figure 1). It becomes necessary to invoke very fast rates of evolution during the early stages of photosynthesis. One might think that the reason why D1 and D2 are evolving so slowly is because water oxidation places a penalty on mutation rates; therefore, one would expect that the anoxygenic reaction center proteins are evolving faster than Photosystem II proteins. In comparison, the L and M of *Chloroflexus aurantiacus* share 24.7% identity between each other and those of *Blastochloris viridis* share 21.9% and are also evolving at a slow rate, albeit just slightly faster than D1 and D2. This is assuming that the phylum Chloroflexi and the phylum Proteobacteria had already appeared, at the very least, around the time of the Great Oxygenation Event. Thus, at that time, they should have carried highly divergent L and M characteristic of their own phylum. If we take into account the large phylogenetic distance among reaction center proteins and their rates of evolution, then the appearance of reaction center proteins around 3.8 billion years ago is likely. Not only that, but if the ancestral Photosystem II before the split of D1 and D2 could have carried out some inefficient water oxidation, then primordial forms of oxygenic phototrophic bacteria 3.2 billion years ago, or before, is perfectly feasible. A detailed analysis on the evolutionary rates of reaction center proteins will be published elsewhere.

There are two main implications derived from the discussions in here. First, if photosynthesis is an ancient process originating 3.8–3.5 billion years ago, this should place its origin near the root or at the root of the bacterial tree of life. It means that photochemical reaction centers were crucial in the development of bacterial bioenergetics systems and were not just merely appended to it. The second ramification is the massive loss of phototrophy throughout the bacterial tree of life, regardless of how pervasive horizontal gene transfer has been. Unlike the trees for Rubisco (Tabita et al., 2008), Nitrogenase-like proteins (Raymond et al., 2004), or Cytochrome *bc*_1_/*b*_6_*f* complexes (Nitschke et al., 2010), which are quite leafy; the trees of reaction center proteins look like a London Plane tree after the end of the winter: very long branches and only a few leaves left. The long branches summed to relatively slow rates of evolution suggest that the vast majority of phototrophs have gone extinct and that many still remain to be discovered.

## Author contributions

The author confirms being the sole contributor of this work and approved it for publication.

### Conflict of interest statement

The author declares that the research was conducted in the absence of any commercial or financial relationships that could be construed as a potential conflict of interest.
